# Supplementary material for: Radiolysis-Assisted Direct Growth of Gold-Based Electrocatalysts for Glycerol Oxidation
Source: Nanomaterials (Basel). 2023 May 23;13(11):1713. doi: 10.3390/nano13111713 (PMC10254145; doi:10.3390/nano13111713)
Supplement: Supplementary file 1 [file nanomaterials-13-01713-s001.zip › nanomaterials-2386248-supplementary.pdf]

Supplementary Materials

Photographs

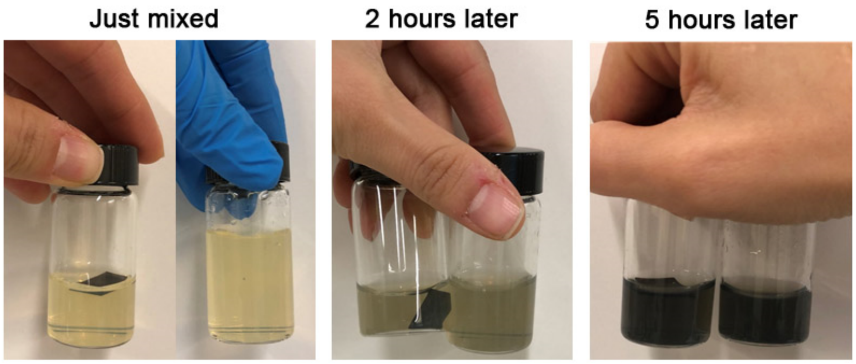

Figure S1. An attempt to synthesize Au-Ag bimetallic particles using NaCit as a stabilizing agent: aggregation of particles occurred in the reaction mixture containing [$AuCl_4^-$] and [Ag^+] regardless of the presence of carbon paper.

Conversion of potential values from MOE to RHE scale

Conversion of potential scale was done according to Nernst relationship E (vs RHE) = E (vs MOE) + ΔE. Experimentally, ΔE = 0.946 V (at 25 °C) in 1 M NaOH according to calibrating curve shown in **Figure S2**. In the calibration measurement, the reference electrode MOE was connected to the cell with a Pt plate as the working electrode and a Pt mesh as the counter electrode in H_2-saturated 1 M NaOH. CV scans were performed at 1 mV·s^{-1}. The CV curve intersects the I=0 axis at two points, whose average gives a thermodynamic potential of the reactions related to H_2-evolution (HER) and H_2-oxidation (HOR).

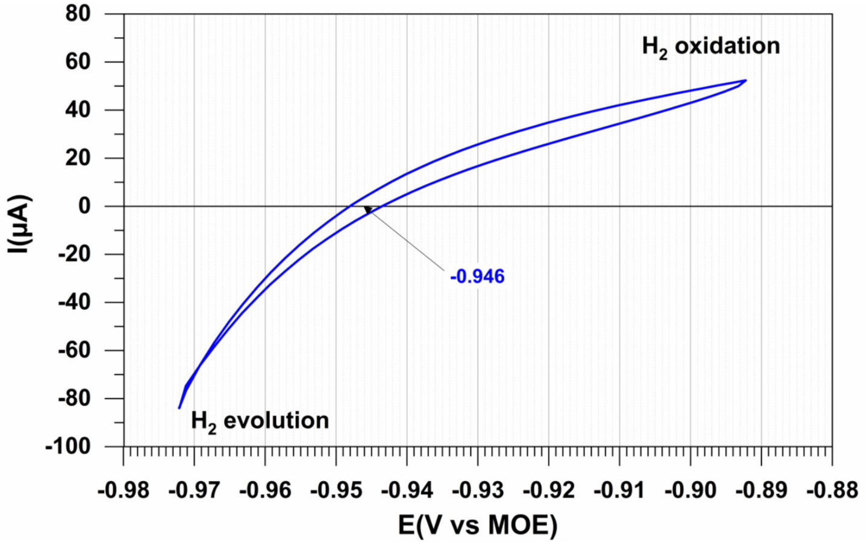

Figure S2. CV for the calibration of the MOE reference electrode (1 M NaOH, 1 mV s^{-1}, 25 °C, unstirred, Pt as working and counter electrodes)

Energy Dispersive X-ray (EDX) Spectroscopy

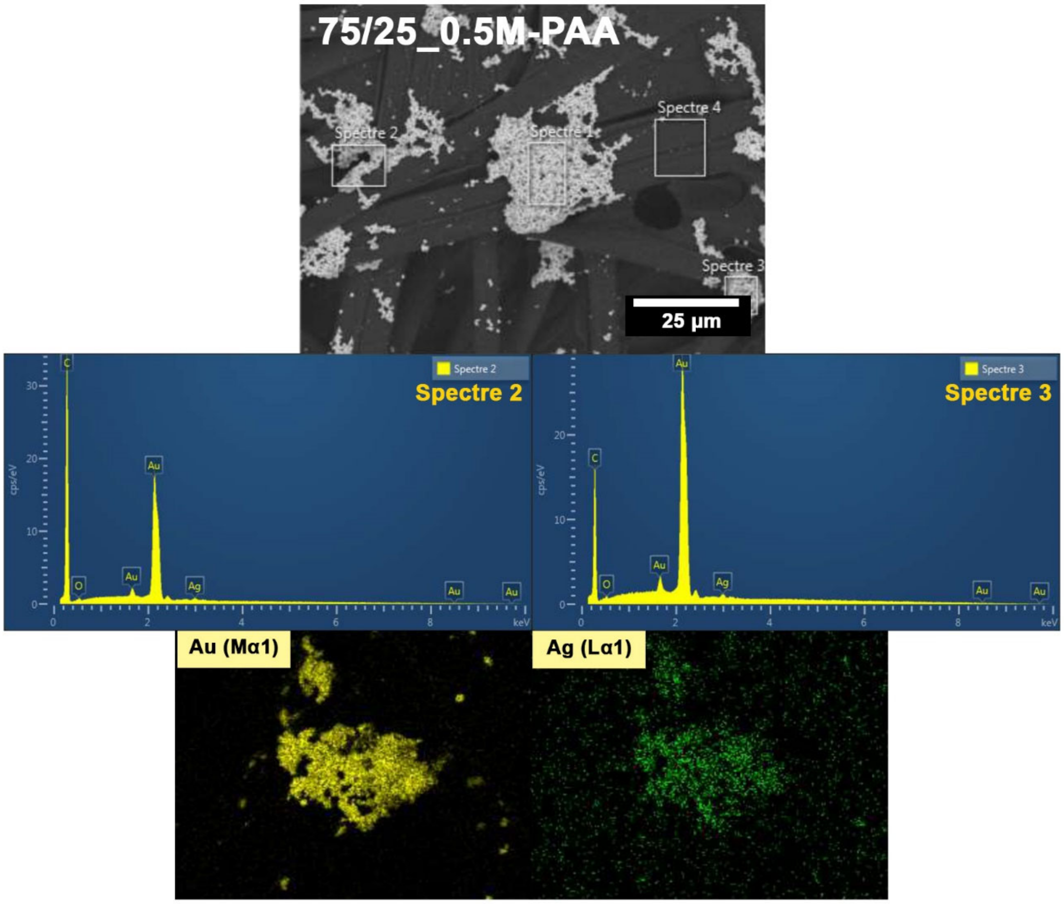

Figure S3. EDX spectra on the labeled zones on SEM micrograph of the bimetallic gamma sample Au_{75}/Au_{25}-0.5M-PAA.

Table S1. Atomic fractions of Au, Ag, C and O (among other trace contaminants) empirically defined by EDX measurements of Au_{75}/Au_{25}-0.5M-PAA on the labeled zones in **Figure S3**.

Analyzed zone	Au at. %	Ag at. %	C at. %	O at. %
Spectre 2	7.67	0.41	90.83	1.10
Spectre 3	18.07	0.88	79.79	1.26

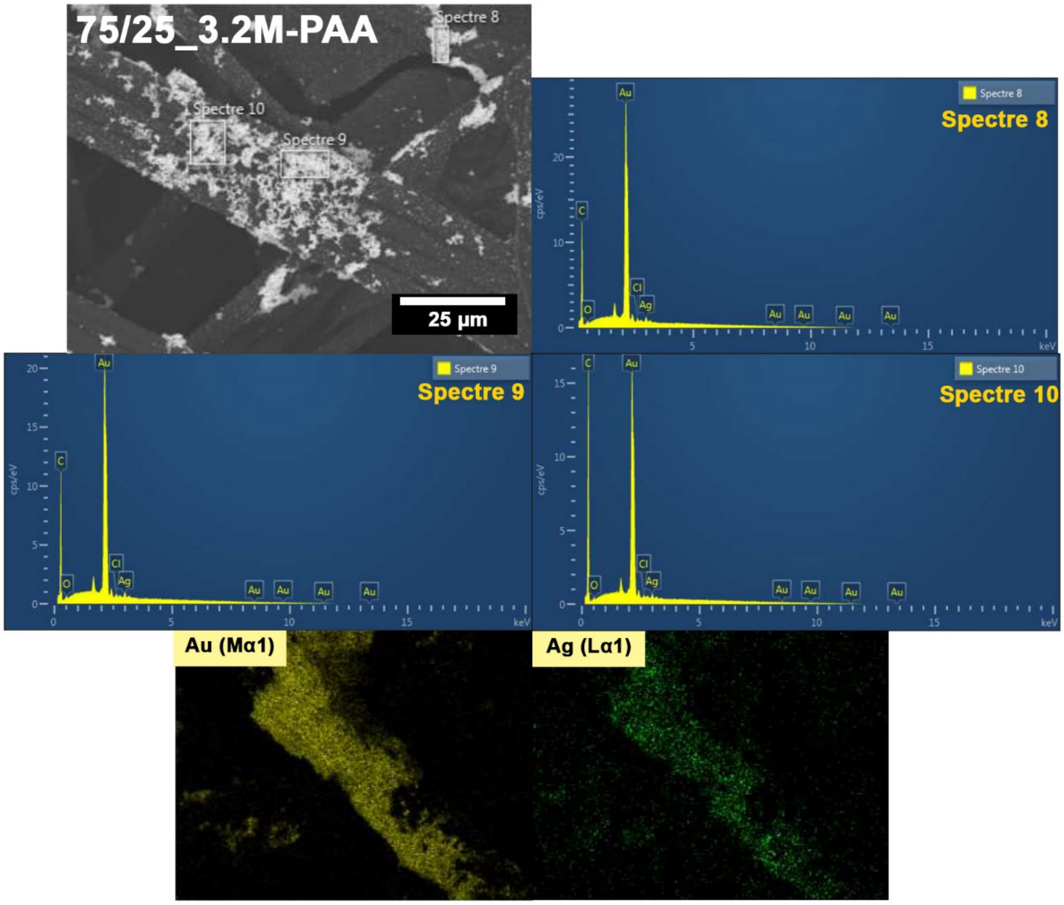

Figure S4. EDX spectra on the labeled zones on SEM micrograph of the bimetallic gamma sample Au_{75}/Au_{25}-3.2M-PAA.

Table S2. Atomic fractions of Au, Ag, C and O (among other trace contaminants) empirically defined by EDX measurements of Au_{75}/Au_{25}-3.2M-PAA on the labeled zones in **Figure S4**.

Analyzed zone	Au at. %	Ag at. %	C at. %	O at. %
Spectre 8	15.78	1.07	81.24	1.28
Spectre 9	14.26	0.96	82.89	1.36
Spectre 10	9.22	0.60	88.99	0.95

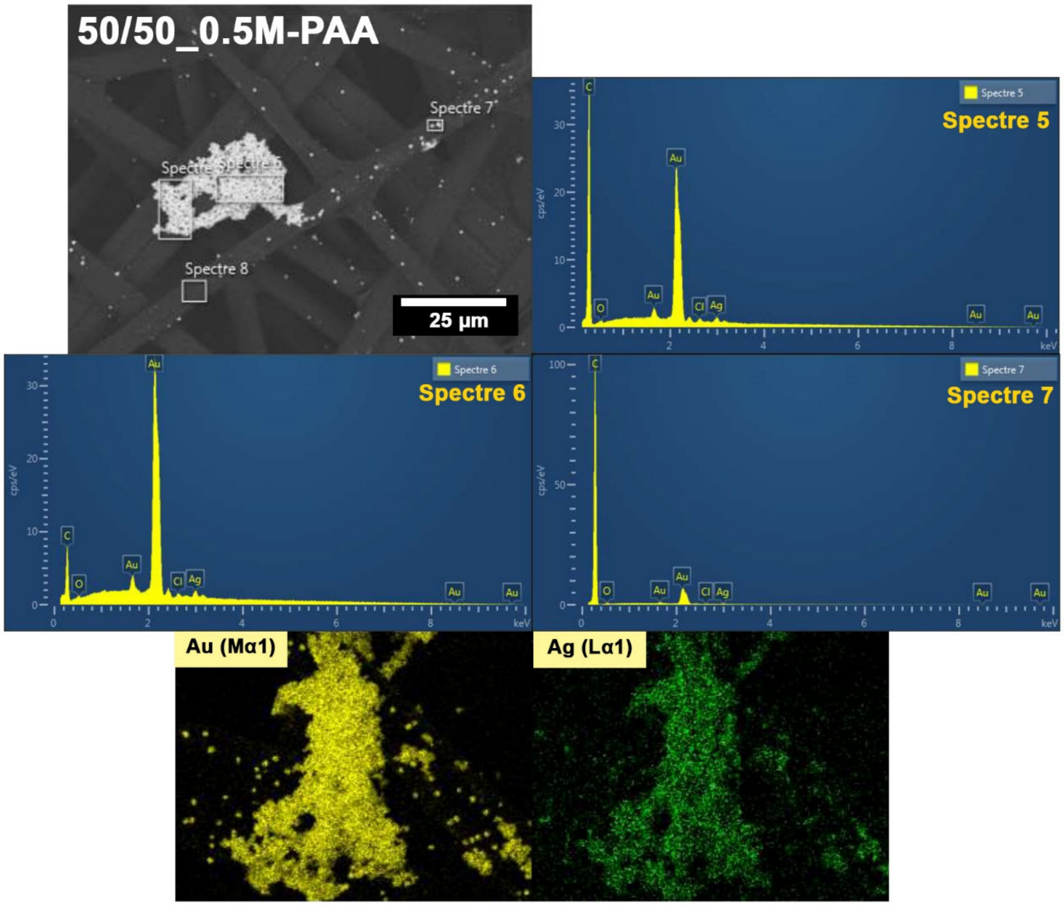

Figure S5. EDX spectra on the labeled zones on SEM micrograph of the bimetallic gamma sample Au_{50}/Au_{50}-0.5M-PAA.

Table S3. Atomic fractions of Au, Ag, C and O (among other trace contaminants) empirically defined by EDX measurements of Au_{50}/Au_{50}-0.5M-PAA on the labeled zones in **Figure S5**.

Analyzed zone	Au at. %	Ag at. %	C at. %	O at. %
Spectre 5	9.02	0.88	88.49	1.07
Spectre 6	30.51	2.64	64.01	1.50
Spectre 7	1.65	0.18	97.12	0.98

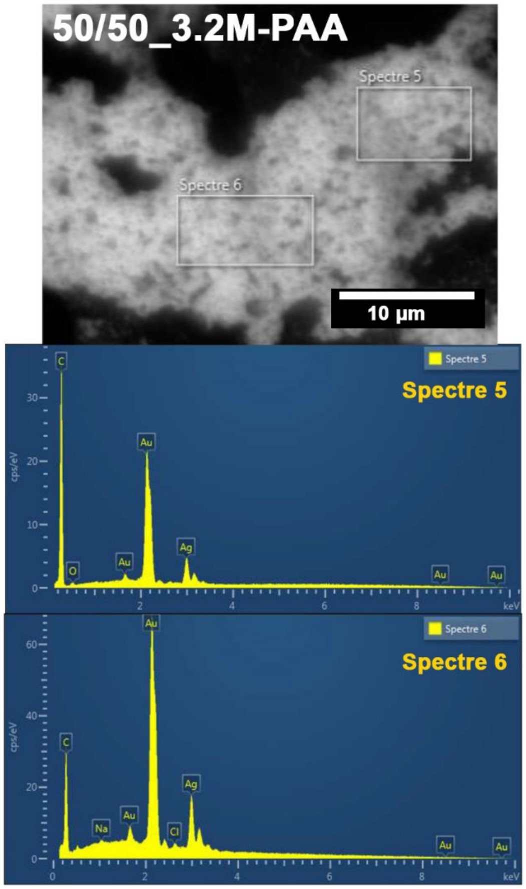

Figure S6. EDX spectra on the labeled zones on SEM micrograph of the bimetallic gamma sample Au_{50}/Au_{50}-3.2M-PAA.

Table S4. Atomic fractions of Au, Ag, C and O (among other trace contaminants) empirically defined by EDX measurements of Au_{50}/Au_{50}-3.2M-PAA on the labeled zones in **Figure S6Error! Reference source not found.**.

Analyzed zone	Au at. %	Ag at. %	C at. %	O at. %
Spectre 5	8.56	4.89	85.10	1.45
Spectre 6	20.01	13.53	65.32	0.42

X-ray Photoelectron Spectroscopy (XPS)

XPS survey spectra.

The double peaks from the Au 4f and 4d orbitals were observed at 80-90 eV and 330-360 eV range, respectively. The presence of silver is supported by the 3d and 3p orbitals in the range of 365-375 eV and 570-605 eV, respectively. The carbon substrate generates XPS signals of C 1s and O 1s in the range of 282-287 eV and 530-535 eV, respectively. NaCl residues are observed with low-intensity peaks at 199 (Cl 2p), 498 ($KL_{23}L_{23}$ of Na), 1072 (Na 1s) eV as contamination, the sodium possibly coming from the PAA purchased as a sodium salt and chlorine from a possible ion exchange reaction with $KAuCl_4$.

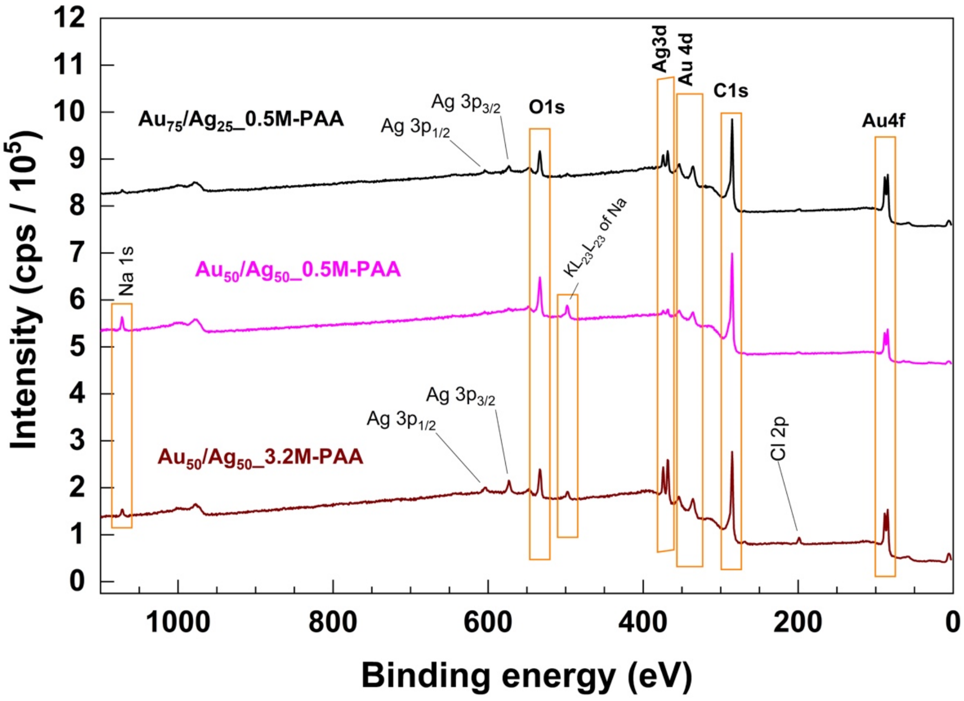

Figure S7. Survey XPS spectra recorded between 0 and 1100 eV for bimetallic Au-Ag samples grown directly onto carbon paper during gamma radiolysis: Au_{75}/Ag_{25}-0.5M-PAA (black curve), Au_{50}/Ag_{50}-0.5M-PAA (magenta curve), Au_{50}/Ag_{50}-3.2M-PAA (brown curve).

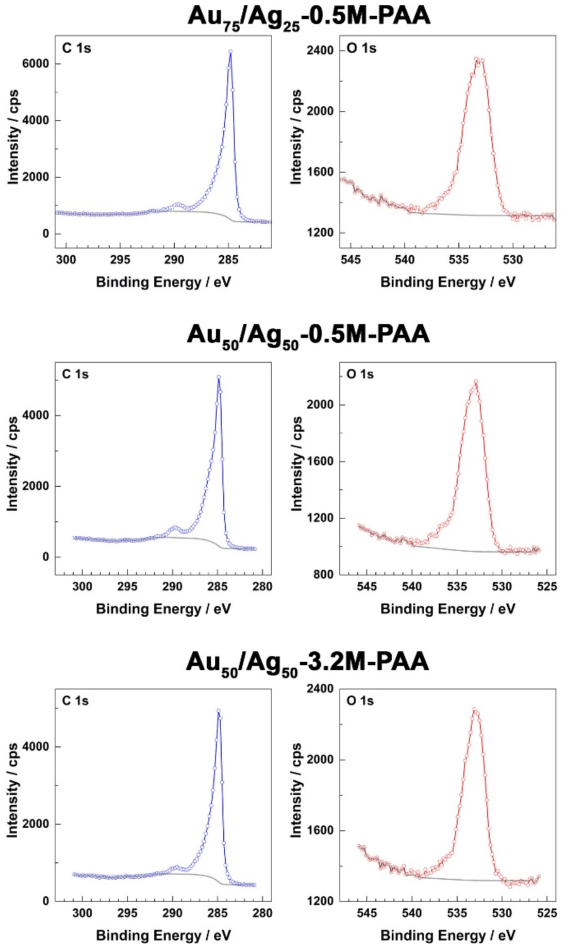

Figure S8. High-resolution XPS spectra recorded for C 1s and O 1s spin-orbitals for the bimetallic gamma-synthesized samples Au_{75}/Au_{25}-0.5M-PAA, Au_{50}/Au_{50}-0.5M PAA and Au_{50}/Au_{50}-3.2M PAA.

Table S5. Deconvolution results of high-resolution XPS spectra of Au_{75}/Au_{25}-0.5M-PAA

Spin-orbital component	Position / eV	FWHM / eV	Atomic fraction / %
Au $4f_{7/2}$	84.4	0.8	3.2
Ag $3d_{5/2}$	368.2	1.1	1.5
Au $4d_{5/2}$ plasmon	370.6	2.2	0.2
C 1s	284.8	1.0	82.1
O 1s	533.4	2.7	13.0

Table S6. Deconvolution results of high-resolution XPS spectra of Au_{50}/Au_{50}-0.5M-PAA

Spin-orbital component	Position / eV	FWHM / eV	Atomic fraction / %
Au $4f_{7/2}$	84.3	0.8	1.8
Ag $3d_{5/2}$	368.2	1.2	0.3
Au $4d_{5/2}$ plasmon	371.4	2.4	0.1
C 1s	284.9	1.2	80.5
O 1s	532.9	3.0	17.3

Table S7. Deconvolution results of high-resolution XPS spectra of Au_{50}/Au_{50}-3.2M-PAA

Spin-orbital component	Position / eV	FWHM / eV	Atomic fraction / %
Au $4f_{7/2}$	84.4	0.8	3.1
Ag $3d_{5/2}$	367.9	1.3	2.9
C 1s	284.9	1.1	79.5
O 1s	533.1	2.7	14.5

X-ray Diffraction analysis

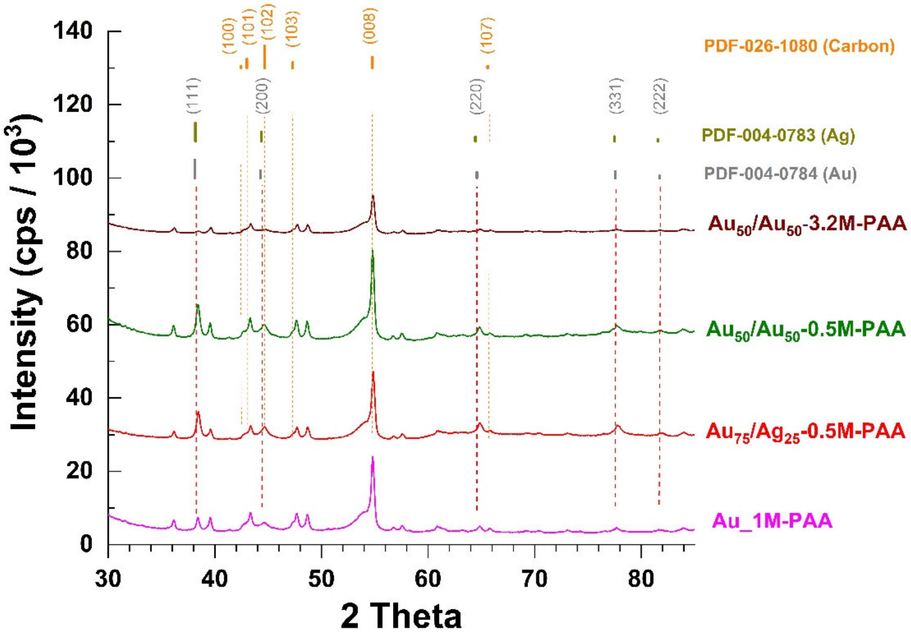

Figure S9. XRD pattern recorded for monometallic Au_1M-PAA and bimetallic samples Au_{75}/Ag_{25}-0.5M-PAA (red curve), Au_{50}/Ag_{50}-0.5M-PAA (green curve), Au_{50}/Ag_{50}-3.2M-PAA (brown curve) between 2θ angles of 30° and 85°. Reference XRD patterns of the *fcc* Au (JCPDS 00-004-0784, gray bars), the *fcc* Ag (JCPDS 00-004-0783, green bars) and the carbon (JCPDS 00-026-1080, orange bars) are shown for comparison. Red and orange dashed lines are drawn for easier comparison of the metal and carbon peaks, respectively.

Cyclic voltammetry of underpotential deposition of lead

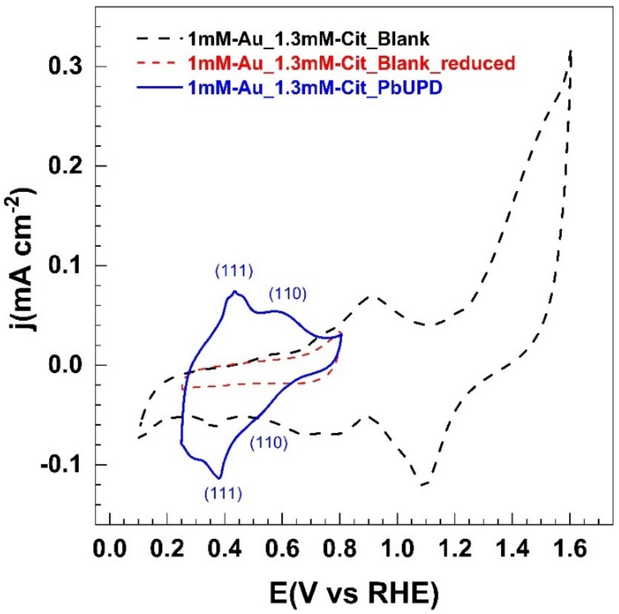

Figure S10. Cyclic voltammograms recorded for Au_1.3mM-Cit at 25 °C at 50 mV·s^{-1}: black dashed curve – blank record in 1 M NaOH solution between 0.1 and 1.6 V vs RHE; red dashed curve – blank record in 1 M NaOH solution between 0.25 and 0.8 V vs RHE; blue solid curve – Pb UPD records in 1 M NaOH solution with 1 mM $Pb(NO_3)_2$ between 0.25 and 0.8 V vs RHE.

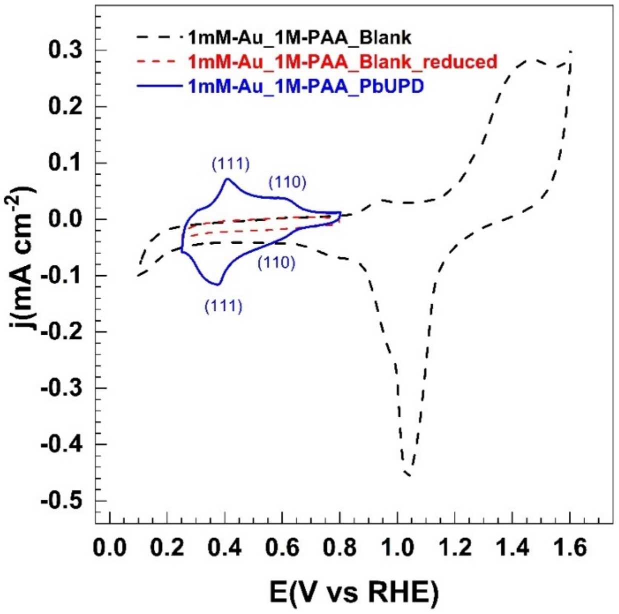

Figure S11. Cyclic voltammograms recorded for Au_1M-PAA at 25 °C at 50 mV·s^{-1}: black dashed curve – blank record in 1 M NaOH solution between 0.1 and 1.6 V vs RHE; red dashed curve – blank record in 1 M NaOH solution between 0.25 and 0.80 V vs RHE; blue solid curve – Pb UPD records in 1 M NaOH solution with 1 mM $Pb(NO_3)_2$ between 0.25 and 0.8 V vs RHE.

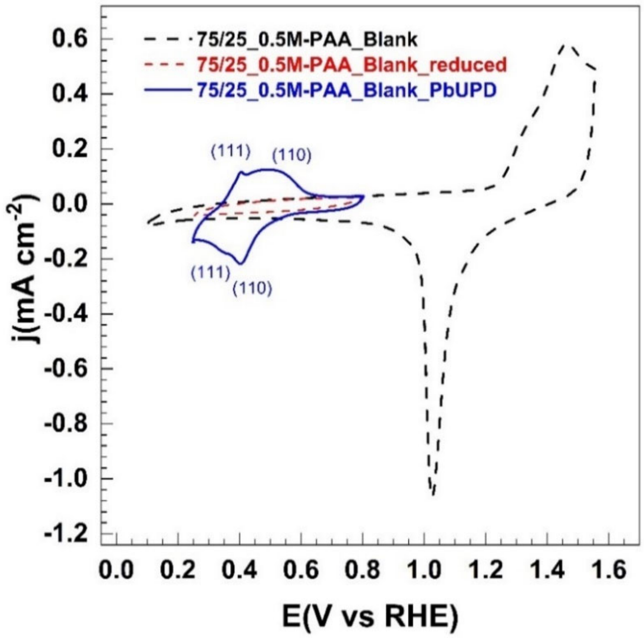

Figure S12. Cyclic voltammograms recorded for Au_{75}/Au_{25}-0.5M-PAA at 25 °C at 50 mV·s^{-1}: black dashed curve – blank record in 1 M NaOH solution between 0.1 and 1.55 V vs RHE; red dashed curve – blank record in 1 M NaOH solution between 0.25 and 0.8 V vs RHE; blue solid curve – Pb UPD records in 1 M NaOH solution with 1 mM $Pb(NO_3)_2$ between 0.25 and 0.8 V vs RHE.

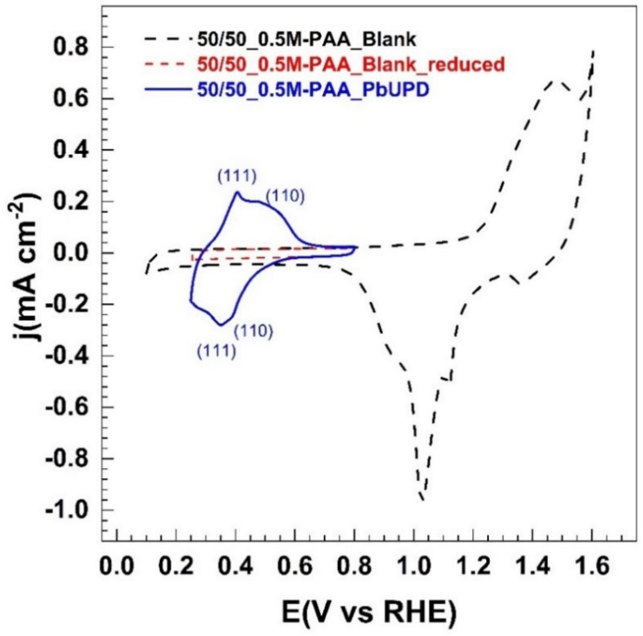

Figure S13. Cyclic voltammograms recorded for Au_{50}/Au_{50}-0.5M-PAA at 25 °C at 50 mV·s^{-1}: black dashed curve – blank record in 1 M NaOH solution between 0.1 and 1.55 V vs RHE; red dashed curve – blank record in 1 M NaOH solution between 0.25 and 0.8 V vs RHE; blue solid curve – Pb UPD records in 1 M NaOH solution with 1 mM $Pb(NO_3)_2$ between 0.25 and 0.8 V vs RHE.

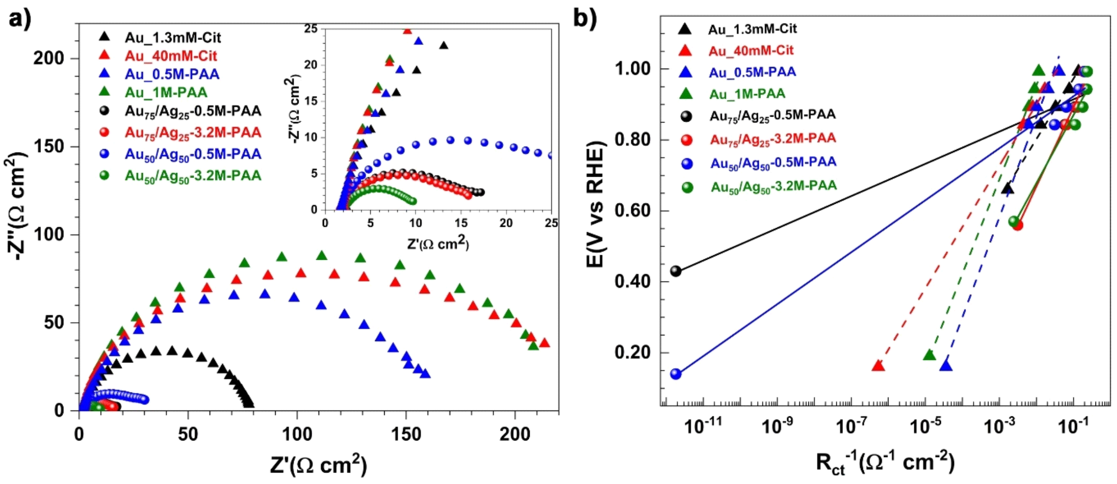

Figure S14. Nyquist plots recorded at applied potentials of 0.84 V vs RHE in aqueous solution containing 1 M of NaOH and 0.1 M of glycerol for monometallic samples (▲) Au_1.3mM-Cit, Au_1M-PAA and bimetallic samples (●) Au_{75}/Ag_{25}-0.5M-PAA, Au_{75}/Ag_{25}-3.2M-PAA, Au_{50}/Ag_{50}-0.5M-PAA and Au_{50}/Ag_{50}-3.2M-PAA at 25 °C. (b) Plot of applied potential as a function of reverse R_{ct} (E vs R_{ct}^{-1}) and their fittings.

Cyclic voltammetry

Characterization by cyclic voltammetry (CV) scans was carried out in a three-electrode cell in alkaline conditions. 1 M aqueous solution of NaOH was used as an electrolyte. A double junction Mercury-mercury oxide electrode (MOE) was purchased from Origalys France and utilized as a reference electrode. All CV graphs were reported with potential values converted to the reversible hydrogen electrode (RHE) scale. The conversion was done according to Nernst relationship E (vs RHE) = E (vs MOE) + ΔE. Experimentally, ΔE = 0.946 V (at 25 °C) in 1 M NaOH according to calibrating curve shown in **Error! Reference source not found.**. In the calibration measurement, the reference electrode MOE was connected to the cell with a Pt plate as the working electrode and a Pt mesh as the counter electrode in H_2-saturated 1 M NaOH. CV scans were performed at 1 mV·s^{-1}. The CV curve intersects the I=0 axis at two points, whose average gives a thermodynamic potential of the reactions related to H_2-evolution (HER) and H_2-oxidation (HOR).

A 1×0.5 cm rectangular piece of the as-prepared CP was cut out to form the working electrode for the CV measurements. Note that only half of this piece (0.5×0.5 cm) was immersed in the solution and served as a working area, providing 0.25 cm^2 of the geometrical surface area that received the deposition. The real surface area of the 3D structure of microfibers was not taken

into account. The second half of the CP was left for the fixation to the wire. A glassy carbon plate of 12.4 cm^2 was used as a counter electrode. The scan rate of the CV measurements was set to 50 $mV \cdot s^{-1}$ between 0.1 and 1.6 V vs. RHE. CV measurements are referred to as a *blank* CV since the scans were performed in the absence of glycerol.

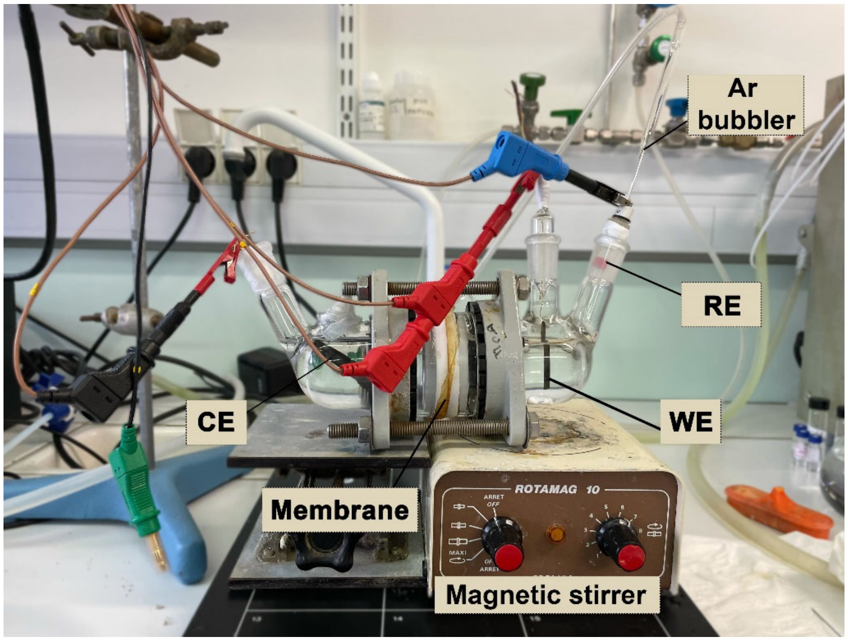

Figure S15. An H-type cell composed of right and left compartments for chronoamperometric measurements of the working electrode (labeled as WE) in glycerol electrooxidation. The working electrode is the carbon paper piece after deposition of Au or Au-Ag. It is located on the right compartment with RE, reference electrode that is Hg|HgO|NaOH 1 M MOE, and Ar bubbler, a glass tip of the inserted argon bubbling tube. A counter electrode (labeled as CE) that is a glassy carbon plate inserted on the left side compartment. Anion exchange membrane (labeled as membrane) is placed between two compartments.

Table S8. Concentrations (in mM) of the products after chronoamperometry (CA) conducted for 15 and 30 minutes of and corresponding selectivities (in %) for Au and Au-Ag particles deposited on CP during γ-radiolysis.

Sample reference		**Oxalic**	**Tartronic**	**Glyceric**	**Glycolic**	**Formic**
Au_1.3mM-Cit	CA 15 min / mM	0.03	0	0.02	0.20	0.14
	CA 30 min / mM	0.03	0	0.02	0.26	0.15
	Selectivity, %	5.0	0	2.2	**43.1**	**49.7**
Au_1M-PAA	CA 15 min / mM	0.03	0	0.02	0.31	0.18
	CA 30 min / mM	0.03	0	0.03	0.41	0.22

	Selectivity, %	3.3	0	2.2	**45.6**	**48.9**
Au_{75}/Ag_{25}-0.5M-PAA	CA 15 min / mM	0.32	0.01	0.42	7.2	4.5
	CA 30 min / mM	0.57	0.05	0.67	13.8	9.4
	Selectivity, %	1.7	0.10	1.3	**41.1**	**55.8**

Continuation of Table S8

Au_{75}/Ag_{25}-3.2M-PAA	CA 15 min / mM	0	0	0.13	2.8	1.9
	CA 30 min / mM	0.08	0	0	4.6	3.3
	Selectivity, %	0.70	0	0	**41.1**	**58.2**
Au_{50}/Ag_{50}-0.5M-PAA	CA 15 min / mM	0.12	0	0.25	0	4.4
	CA 30 min / mM	0	0	0.29	6.7	6.0
	Selectivity, %	0	0	1.0	**35.3**	**63.7**
